# Perceptions and understandings of pregnancy, antenatal care and postpartum care among rural Lao women and their families

**DOI:** 10.1186/s12884-016-1031-8

**Published:** 2016-08-25

**Authors:** Vanphanom Sychareun, Vathsana Somphet, Kongmany Chaleunvong, Visanou Hansana, Alongkone Phengsavanh, Sisouvanh Xayavong, Rebecca Popenoe

**Affiliations:** 1 Faculty of Postgraduate Studies, University of Health Sciences, Vientiane, Lao People’s Democratic Republic; 2 Faculty of Basic Sciences, University of Health Sciences, Vientiane, Lao People’s Democratic Republic; 3 Department of Neurobiology, Care Sciences and Society, Division of Nursing, Karolinska Institutet, SE-171 77 Stockholm, Sweden

**Keywords:** Traditional birthing practices, Antenatal care, Postpartum care, Cultural beliefs, Low and middle-income country

## Abstract

**Background:**

Lao People’s Democratic Republic (Lao PDR) has the highest maternal mortality rate (MMR) and infant mortality rate (IMR) due to traditional practice and beliefs on pregnancy, delivery and postpartum. The objective of this study was to get a better understanding of cultural beliefs and practices surrounding pregnancy, ANC and postpartum care among rural women in Lao PDR.

**Methods:**

Eight focus group discussions and 52 interviews were carried out with delivered women, husbands, mothers, traditional birth attendants, head villagers, Lao Women’s Union members and healthcare workers, in Khammouane and Champasack provinces in Lao PDR. In order to accurately grasp participants’ perceptions and understandings, content analysis was used to analyze the transcripts.

**Results:**

Most women in the study claimed to have attended ANC, but participants also explained that it was unnecessary to attend ANC and give birth at a clinic if the woman felt healthy. Factors that discouraged ANC attendance and giving birth at clinics included: time and money constraints; the perceived necessity of giving birth on a “hot bed”; the need for “mother-roasting” after giving birth; the belief that preparing for a birth was a bad omen for the birth; the belief that colostrum is unhealthy for the newborn child; and the preference for cutting the umbilical cord with a piece of sharpened bamboo.

**Conclusions:**

Harmful cultural practices such as discarding colostrum should be discouraged; beneficial practices such as family involvement in birthing and keeping a mother warm after birth could be integrated into biomedical practice. Given the prevalence and importance of the cultural understandings we have described in this study, it is clear that proposed changes in cultural practices need to be addressed with sensitivity and that community stakeholders and trusted leaders will need to be involved.

## Background

Lao People’s Democratic Republic (Lao PDR) has the highest maternal mortality rate (MMR) and infant mortality rate (IMR) in Southeast Asia, with 357 maternal deaths per 100,000 live births and 68 infant deaths per 1000 live births [1]. Though these rates are decreasing, the rates of decrease have slowed recently [2].

One explanation for the comparatively high MMR and IMR rates is that as of 2012 62.5 % of births in Lao PDR still took place at home, and only 54.2 % of births were to women who had received antenatal care (ANC) [1]. Those who do not attend ANC and who give birth at home are also more likely to be poor, uneducated, and to live far from health facilities [3–5]. They are also more likely to be from highland or smaller ethnic groups [4] and to adhere to traditional beliefs and practices [6].

All of this suggests that in order to lower the MMR and IMR in Lao PDR a better understanding of pregnancy and birthing practices and attitudes in rural areas is needed. What aspects of traditional care are especially important to rural villagers? Which practices conflict with biomedical understandings and practices, possibly discouraging women and their families from attending health centres, and which practices are harmless and possibly even beneficial? The goal of the study is to gain knowledge about rural Laotian birthing practices and preferences in order to help improve ANC services, encourage villagers to use them, and decrease the MMR and IMR in rural Lao PDR.

Smaller, rural ethnic groups comprise 32 % of the population of Lao PDR [7] but there is relatively little research on traditional birth practices and attitudes among them, hence this study’s focus on focus on the reasoning, practices and attitudes of women and their families during pregnancy, birthing, and the postpartum period.

Previously reported pregnancy practices include, in Saravane province, food taboos so that the baby will not be too large at delivery, and working hard throughout pregnancy in order to facilitate delivery [6]. The vast majority of rural villagers traditionally give birth in their homes [8, 9] though among some smaller ethnic groups (for example the Kry) women go to the forest or to birthing huts to give birth [10, 11]. Common attendants at home births are mothers, husbands, grandmothers, other family members, and traditional birth attendants (TBAs) [12].

A vital practice in many areas is giving birth on a “hot bed” [12–14], also known asso- “mother-roasting” (Lao: *Yu Kam* or *Yu Fai*),whereby mothers lie on a bamboo bed with hot coals under it after birth in order to restore the body’s energy and close off the vaginal opening again [9, 12, 14–16]. Based on a humoral system in which heat is thought to be lost during the “open” state of delivery, the goal of the “hot bed” and all postpartum care is to restore the mother to equilibrium by continued heating of her body [17].

Although these cultural practices around rural birthing in Lao PDR have been described in previous research, there is a lack of in-depth, qualitative research on the perceptions and understandings behind these practices, particularly in the context of attempts to get women to give birth at health facilities.

## Methods

The study is based on interviews and focus group discussions carried out in two provinces, both purposively selected for their ethnic diversity: Khammouane province in central Laos, and Champsack province in southeastern Laos. Within each province two districts were selected also purposively, one ‘intermediate’ and one ‘remote’ according to socio-demographic indicators such as literacy rates and the proportion of the population belonging to socially marginalized groups. The details of the study sites have been described in a previous paper [12].

### Participants

Eight focus groups discussions (FGDs) were carried out with a total of 43 women who had had home deliveries within the past year. Of these 43 women, 12 who had experienced a range of pregnancy outcomes, including obstructed labor and stillbirth, were selected for in-depth interviews (IDIs). Eight of their husbands and eight of their mothers were also interviewed in order to gain a fuller understanding of traditional birth practices. In addition, eight TBAs, six provincial and district healthcare workers, six head villagers and six representatives from the Lao Women’s Union responsible for maternal and child health (MCH) were interviewed.

### Data collection

Data collection took place in December 2008 and January 2009. Newly delivered women and their family members were asked about their feelings around and attitudes to pregnancy; their practices during pregnancy, delivery and postpartum, and constraints they perceived to ANC and delivering at health centers. TBAs, head villagers, representatives of the Lao Women’s Union and healthcare workers were asked about their perceptions of the policies and strategies of the safe motherhood program implemented at the provincial and district levels.

The research team, four trained interviewers and three field assistants from the Faculty of Postgraduate Studies, were recruited and trained to collect information in the study areas. In each FGD, the moderator explained the general topics to be discussed and let participants know that everyone was invited to contribute their ideas. The moderator was assisted by two research assistants, one taking notes and another making observations. The FGDs were recorded using a voice recorder. The details of data collection are explained in more detail in a previous paper [12].

The in-depth interviews were held in a quiet place in the participants’ homes. Each informant was interviewed once and the interviews lasted between 1 and 2 h. FGDs were carried out in the local temple or at the office of the village head. Additional details of the selection of key informants were described in the previous paper [12].

The FGDs and the interviews were carried out in the Lao language or, where needed, in local languages via translators. We tried to build good rapport with women and we provided small towels to women to thank them for their valuable time.

### Data analysis

The recordings as well as notes taken were used to generate transcripts that were in turn, where necessary, translated into Lao. Content analysis was then used to analyze the transcripts. Data analysis was done manually. The investigators read the transcripts many times in order to gain an overview of the material and the other authors then added their own views of the codings and together they reached a consensus. The meaning units were identified and then abbreviated to condensed meaning units. The condensed meaning units were labelled with codes, and different sub- categories were tabulated in order to find different and similar sub-themes.

To enhance credibility of the study’s overall results, triangulation was achieved by using different methods (documentation review, interviews and FGDs) and sources. In addition, field notes, interview transcripts and documented data analysis provide a research trail.

## Results

### Socio-demographic characteristics

The women, their husbands and their mothers in the study were almost all farmers, and were divided evenly between Buddhists and animists. Most had some primary school education but about a third of the women and their husbands, and half of the mothers, were illiterate.

Of the eight TBAs, two were trained medical professionals, one was a medical assistant and the other was a midwife who had worked at the provincial hospital. Of the six others, only two received training at the district level in attending births. Trained or not, most of the eight TBAs had over ten years of experience attending births. Of the 12 head villagers interviewed, aged 35–61 years, all were male but one. All of them were literate, with at least a primary school education. Most were Buddhist, but a few were Christian or Animist.

### Attitudes towards childbearing

Most women reported feeling happy when they were pregnant, mostly because pregnancy and childbirth made them feel valued by their families and their husbands, and acted as cement to their marriages. As one 30-year old woman put it:

… *A child is like a chain connecting the father and the mother. I also worry that if I do not have a baby my husband will divorce me. So when I was pregnant I was very happy*.

One 34-year old husband described his feelings surrounding his wife’s childbearing thus:

*I was very proud to have a child. It secures the reputation of the family and myself because my family is small. I think that in the future*, *I can rely on my child and my children will take care of me and they will grow up and have a prosperous future and I will be proud of them*.

### The experience of pregnancy

Although pregnancy was undeniably a happy occurrence, women noted that one became more tired and more emotional when pregnant. “*The symptoms of childbearing*,*”* one mother of a delivered woman noted, *“are to have dizziness*, *lose one’s appetite*, *and lose weight. Childbearing is torture. It is difficult to walk and sit and stand up due to one’s big abdomen.”*

Some women worried about difficult labor or even death, as well as the future health status of both mother and baby. “*I am concerned about my health in the future*,*”* one woman commented. *“As the old expression goes*, *‘When the baby is inside the womb*, *one is waiting for death.’*

The increase in social value pregnancy conferred upon a woman, however, helped offset the physical and emotional burdens pregnancy entails.

### Traditional antenatal care practices

Most informants were fully aware of the potential risks of pregnancy, including miscarriage, hemorrhage, premature delivery, death of the fetus, and stillbirth and maternal death. A grandmother noted that the health risks during pregnancy include “*exhaustion*, *vertigo*, *and miscarriage*; *all dangers to the fetus. To prevent these*, *pregnant women have to take care of themselves and not work hard”*.

It was believed that women should engage in light housework so that they will have enough energy for labor, as previous research suggested, but that they should avoid heavy labor such as carrying water or working in the rice fields. It was believed that sleeping during the daytime, even if a woman was tired, could lead to difficult labor, retention of the placenta, and jaundice in the baby, so pregnant women were not supposed to take naps during the day.

To relieve women of heavier tasks during pregnancy, relatives and even husbands helped. “*When I was pregnant my husband shared in my daily housework such as cooking*, *farming*, *gardening*, *and washing clothes*,*”* one 18-year old newly delivered woman said. Women also expected their husbands to provide psychological and emotional support.”*I wanted my husband to take care of me and guide me to the hospital*,*”* one 25-year old woman said. *“I did not want my husband to go out socializing and having girls*, *or even drink and smoke during my pregnancy.”* This was especially important because it was thought that pregnant women were easily angered.

Both women and their husbands described how sexual intercourse during pregnancy, especially during the third trimester, could lead to early contractions, miscarriage, injury to the fetus or congenital malformations, preterm labor, prolonged labor, and obstructed delivery. One husband said,

*I did not have sex with my wife during pregnancy starting from the third trimester because it could have damaged the health status of my wife and fetus. Sperm can have a negative effect on the fetus and can cause there to be a lot of lochia [*Lao: *Nam Khao Pa] and bleeding or discharge after delivery [*Lao*: Leud Lay Ok Cha Songkhoat]*.

Only a minority of informants seemed unaware of the potential risks of pregnancy, but all informants were aware of the risks during childbirth, particularly if labor was prolonged. One 32-year old husband commented:

*I know that there is no risk during pregnancy*; *however*, *there are some risks during labor when the labor is prolonged and difficult*, *and [whether this will happen] cannot be known in advance*.

### Attitudes towards and practices of biomedical antenatal care

The majority of women claimed they had attended antenatal care at the health centers and district hospitals, in order to prevent complications during pregnancy and to ascertain that both the mother and baby were healthy. Women who had worries about their health seemed more likely to seek care. “*During pregnancy*, *my wife attended ANC at the district hospital because my wife was not so healthy*, *and she often got sick*,*”* one husband described. *“We wanted to prevent a difficult labor.”*

When women had refused to attend ANC, even against the advice of their husbands or mothers, they said it was because they felt healthy, perceived no risks to their pregnancy, or had no previous experience with childbearing. Other important reasons for not attending ANC were lack of time, lack of money, lack of means of transport, and long distances. Husbands sometimes recommended that their wives receive ANC from a TBA rather than go for ANC at a distant health center that involved considerable transportation costs.

One TBA noted that:

*Most of the pregnant women come to my house for their antenatal visit. I give advice to them that they should take care of themselves during their pregnancy*, *especially by wearing comfortable clothes and not drinking alcohol because of negative health consequences to the baby. I also suggest that they prepare some things for delivery. In addition*, *I advise them to attend ANC at the health facilities more frequently*, *especially during the last trimester.*

TBAs also said that they recommended that a woman go to a health facility if her pregnancy was at risk and if rest and avoidance of hard work had not provided relief, or if the TBA detected an abnormal fetal position. “*If I find a risky case I suggest that they go to the hospital*, *because I do not have the equipment to manage*,*”* one TBA said. *“Especially in case of the mal position/presentation of the baby – I have never assisted birth by myself. I just tell them to go the hospital.”*

Some women said they attended ANC with the mobile outreach teams that come to the villages and offer general health services, but these teams visit villages at most every three months and sometimes as infrequently as twice a year.

### Preparing for labor and delivery

Some informants said that when the birth was approaching they prepared items for childbirth, while others, a minority, feared that advance preparations, especially of baby clothes, could lead to the death of the unborn child. As one 25-year old woman described,

*I did not prepare anything for my baby. I was told by the elders that I did not need to prepare anything in advance for my baby. It is believed that this will cause a stillbirth or death of the fetus in the uterus*.

When preparations were undertaken in advance, husbands, the women’s mothers, or TBAs assembled the necessary items: nappies, baby clothes, rope, ginger, herbal medicine, boiled water, wood/charcoal and a bamboo bed, money, and sarongs as gifts for the TBAs. The rope is used for the woman to pull on or hang on during labor, the herbal medicines and ginger are boiled and drunk after delivery and during the postpartum period in order to stimulate the breast milk, and the bamboo bed and wood or charcoal are so that the woman can lie on a “hot bed” after delivery.

Some TBAs considered it the woman’s responsibility to prepare a razor blade or sharp piece of bamboo for cutting the umbilical cord as well as other items, while others prepared these things themselves. TBAs who had undergone training at the district health office were likely to have delivery kits with them. One TBA described her usual preparations,

*For birth preparedness*, *I prepare soap*, *gloves*, *candles and magic water* (Lao: *Nam Mon*) *to blow and spray on the mother in case of a difficult delivery. For cutting the umbilical cord I prepare the sharp bamboo piece because I think it is clean enough*, *and I have never used the razor blade to cut the cord*, *because I am afraid the baby will get umbilical tetanus*.

### The postpartum period

Traditionally TBAs cut the umbilical cord using a sharpened piece of bamboo, but today many use a razor blade. Some TBAs explained how they wash the razor blade with alcohol, and use a piece of black or white string to tie the umbilical cord. As one TBA described,

*I used to cut the umbilical cord by using a sharp bamboo piece because I had no delivery kit. I think it was clean and safe*, *and it was easy and available in our community. In my experiences there were no cases of tetanus of the umbilical cord from using a sharp piece of bamboo to cut the umbilical cord*.

After the cutting of the cord, the baby is bathed and then placed on a bamboo bed near the mother. The mother is then prepared for the “hot bed” by having water from a traditional healer blown on her and having tight black and red cotton tied around her wrists, ankles and neck in order to prevent bad spirits from entering her.

With a fire lit beneath it, the “hot bed” is thought to strengthen the woman’s health and accelerate contraction of the uterus. Salt is put on the fire to prevent skin rashes in both newborns and mothers, to create strong eyes in newborns and to generally aid the health of the mother and the newborn. During the time she lies on the hot bed the woman also takes hot baths and drinks hot water. As one 50-year old mother of a newly delivered women described,

*According to the ritual practice*, *postpartum women had to sit on a banana leaf with salt on it for about 40 min in order for the wounds [of childbirth] to dry more quickly. After that*, *women had to take a hot bath with herbal medicines before lying over the hot fire*, *drinking hot herbal medicines mixed with water -- about 4 pots --*, *and taking hot baths early every morning without cleaning the skin for two weeks*.

It was also reported that fish net is put on the ceiling in the room where the newly delivered woman lies in order to prevent spirits from taking the souls of mothers and newborns.

Commonly husbands bury the placenta deep down in the earth under the floor of the house. It is considered dirty and therefore cannot simply be thrown away. A fire is lit around the place where the placenta is buried in order to prevent spirits and animals from reaching it. If spirits and animals touch the placenta, it is believed that it may cause the lochia to dry up, may inflict diarrhoea on the child, or even cause neonatal death. One husband said:

*I buried the placenta under the floor near the stairs and made a fire near the place. It is believed that if we bury the placenta far from a house*, *the child will move away. Making the fire is [also] related to the belief that the umbilical cord will fall off quickly*.

In fear that the placenta might be dragged out of its burial place by some scavenging animals, some people buried the placenta in a hilly place and did not turn left or right when they buried the placenta.

Some TBAs said that they encouraged mothers to nurse their newborns right after delivery and to give them colostrum. One said,

*After delivery I did not suggest that they give any food or drink to the baby*, *but I suggested that they let the baby nurse at the mother’s breast in order to stimulate lactation and make the uterus contract*.

More commonly, however, women practiced the traditional custom of not giving the newborn colostrum, and waiting to nurse until breast milk comes in. They gave the newborn water to quench its thirst instead, sometimes boiled. One TBA said,

*After delivery I suggested that they give water or honey to the baby to drink by using clean cotton soaked in water or honey and softly pressed to the mouth of baby because the breastfeeding was not ready from mother yet*.

It was also thought that if a baby did not get water soon after birth, it could get jaundice and conjunctivitis. Some informants explained that if you give a newborn water, he will grow up to be clever.

## Discussion

The study reveals that the majority of villagers have considerable knowledge about the potential risks of pregnancy and childbirth, although a minority did not. Villagers also, however, had some traditional beliefs that may work against their seeking adequate ANC, as well as practices surrounding delivery and postpartum that they consider vital to health but that are hard to maintain if giving birth at a health centre.

In contrast to the findings of one earlier study [6], both women and men in our study claimed to be well aware that working too hard during pregnancy could lead to complications, as could having a fetus with an abnormal presentation. Miscarriage and stillbirth, fetal abnormalities, and the risk of hemorrhaging before, during and after childbirth were all known risks in any pregnancy. Perhaps messages that the health centers are trying to teach have now reached villagers, although this does not always mean that they have fully incorporated these ideas into their practices.

Although only half of pregnant women in Lao PDR attended ANC according to a recent survey [1], a majority of women in this study claimed that they attended ANC. The factors that discouraged women from attending ANC were feeling healthy as well as the constraints of time, money, and transportation. This is clearly an area where education could be stronger, alerting women and their families to risks in pregnancy that are not always perceptible to the woman herself. In addition, efforts could be increased to make ANC more accessible to women, via more frequent visits by mobile teams, for example.

Only a few of the women in this study delivered at a hospital. As with decision-making around attending ANC, if women and their families perceived a pregnancy to be low-risk, women and their families did not see the rationale for spending money and time to give birth at a health center, and gave birth at home instead [18].

Traditional rituals surrounding pregnancy, birth and postpartum were considered vital to the woman and the baby’s health, as in previous research [19–22]. A number of cultural practices, including using magic water during delivery and tying strings on women’s wrists to ward off spirits, are not harmful and could be incorporated into ANC and delivery at health centers if this would make women more willing to use these services.

As previous studies indicated [9, 12, 14] lying on a hot bed after delivery and during the postpartum period was considered vital to the success of childbirth and to the healing process of the postpartum period by all we spoke with. These are practices that are not currently possible at health centers, and that no doubt discourage women and their families strongly from making the effort to give birth there. In that this clearly is a crucially important practice for villagers, one recommendation following from this study is that hot beds could be incorporated into health center practices in some form.

Two traditional practices are potentially harmful: using unsterilized bamboo to cut the umbilical cord, and not giving colostrum to newborns. Although TBAs have been instructed to use a sterilized razor to cut the umbilical cord, many felt that a piece of sharpened bamboo worked just as well. Either a way could be found to teach TBAs to sterilize bamboo pieces, or a way should be found to discourage this practice. A previous study [23] also found that women did not allow newborns to nurse until milk came in after a few days. Teaching women that immediate nursing after birth is protective and healthy for their children, and that colostrum is not bad, should be a priority. But culturally sensitive ways must be found to get this information across in a convincing way. These could include using women who already have authority in the village to convince others, media campaigns showing newly delivered women from targeted ethnic groups breastfeeding their newborns directly after delivery, and engaging women’s groups to teach about the subject.

For those we spoke with, preserving the well-being and life of a woman and her child, throughout pregnancy, birth and the postpartum period was, naturally, of vital importance, but they considered other practices than biomedical ones key to insuring this. The best way to increase survival for both mothers and newborns is not to try to force alien practices on women and their families, but to adapt biomedical knowledge and procedures to the value systems and ingrained practices of traditional villagers. Since pregnancy, antenatal care, and postpartum clearly involve husbands and other family members, it is also important that education and services be directed at all, not just women.

This study’s strengths include the high number of participants for a qualitative study; the inclusion of husbands, mothers, TBA’s and other villagers; and the depth and breadth of information obtained from these numerous sources. As the IDI’s and FGD’s turned up largely similar information, and since towards the final IDI’s and FGD’s no new knowledge was found, we feel that the study reached data saturation.

Our study has some limitations. First our findings cannot be generalized to rural Lao women outside Khammouane and Champassack provinces as they are not necessarily representative of the whole country. The key informants were comprised only of women who had experienced a home delivery. The possible influence of male researchers on female participants during data collection and the possibility of social desirability bias might be occurred.

## Conclusion

This study sheds light on rural understandings of pregnancy, antenatal care and postnatal care in the socio-cultural context of Lao PDR as the country tries to spread safer antenatal care, childbirth, and postnatal care to rural populations. Changing women’s attitudes may take some time, but creative campaigns could be developed to help women change the traditional practice of discarding colostrum; beneficial practices such as family involvement in birthing could be integrated into biomedical care; and harmless practices perceived as important such as keeping a mother warm after birth could be integrated into biomedical practice. It is important to alert women and their families to the fact that not all pregnancy risks can be easily detected by the woman herself, or even by TBA, so that ANC is important even when a woman feels healthy. Screening for high risk pregnancies and early referrals to hospitals is also important. Women and their families could be encouraged to make the most biomedically prudent preparations, while perhaps continuing to avoid some preparations that impinge less on the safety of the mother and child, out of respect for cultural traditions. Finally, mobile maternity services might be a solution for women who have trouble getting to a health center.
